# Early Stage Finding of an Immune-Enhanced Genetic Subtype of Nonsmall Cell Lung Cancer Related with T-Cell Depletion

**DOI:** 10.1155/2022/6765997

**Published:** 2022-10-14

**Authors:** Ying yang, Qibin shen, Haijun dong, Tiancheng liu, Shunli dong, Dong li

**Affiliations:** ^1^Department of Thoracic Surgery, Huzhou Central Hospital, Huzhou 313000, China; ^2^Huzhou Key Laboratory of Molecular Medicine, Huzhou Central Hospital, Huzhou 313000, China

## Abstract

**Background:**

Molecular categorization of lung cancer in medical care is becoming increasingly important on a regular basis. One of the molecular classifications of NSCLC (early-stage NSCLC) supports that tumors of different biological varieties differ in terms of their genomes and clinical characteristics.

**Methods:**

Based on published immune cell signatures and early-stage NSCLC gene expression data including cancer genome maps, we used consensus cluster analysis to identify immune molecular subtypes associated with early-stage NSCLC expression subtypes. These subtypes were correlated with early-stage NSCLC expression subtypes. Next, applying a wide range of statistical techniques, we evaluated the link between molecular subtypes and clinical features, immunological microenvironment, and immunotherapy reactivity. Molecular subtypes were defined as a classification of cancerous cells.

**Results:**

Multiple RNAseq cross-platform datasets of immune genes were used to identify two molecular subtypes (C1 and C2) of NSCLC, with C1 showing a more favorable prognosis than C2. The results were validated on the CSE datasets. In terms of clinical characteristics, C2 subtype samples with a worse prognosis showed a more advanced tumor stage and higher mortality. C2 showed immuno-infiltrative characteristics but had depletion of T-cells. Biological functions such as EMT were enriched on C2. A low TIDE score in C1 indicated that C1 samples could benefit from taking immunotherapy. C2 were more susceptible to standard chemotherapeutic treatments such paclitaxel, cisplatin, sorafenib, crizotinib, and erlotinib.

**Conclusion:**

According to our findings, early-stage NSCLC patients may benefit from receiving treatment with immune checkpoint blockade therapy.

## 1. Introduction

Lung cancer is one of the primary reasons leading to cancer death in the world [1]. It is predicted that the incidence of lung cancer would continue to climb in China due to the country's rapidly growing and aging population as well as increased environmental pollution [2]. Histological classification is what differentiates nonsmall cell lung cancer or NSCLC from small cell lung cancer, which accounts for at least 80 percent of all cases of lung cancer. NSCLC and small cell lung cancer are the subtypes of lung cancer. Patients who are diagnosed with lung cancer at an early stage have, in general, a better prognosis than those at a more advanced stage. Significant variation in clinical prognosis is present even among early-stage NSCLC patients with similar macroscopic clinical characteristics, highlighting the need of a deeper understanding about the molecular mechanisms of action [3].

In recent years, new research possibilities in the field of tumor immunology have emerged as a result of the rapid development of high-throughput genomic technologies as well as the rise of bio-informatics tools [4]. It is feasible to obtain an estimate total number of immune cells in the body according to the fact that the gene expression patterns of various cell types are distinct from one another. Researchers have begun probing into the topography of immune cell infiltration based on data obtained from molecular techniques such as sequencing and gene chip analysis. One could view this as a future direction of research. A variety of different bio-informatics methods have been developed in order to accomplish the goal of detecting the stable abundance and relative proportion of immune cell subpopulations present in samples taken from tumorous tissues. To name a few examples, these tools include MCP-counter [5], ESTIMATE [6], and CIBERSORT [7]. The creation of an early-stage staging system for NSCLC based on the features of tumors and the immune responses to those tumor-infiltrating immune cells via such a method is believed to be promising, and future research on immune-related critical regulatory genes and molecular pathways would help to achieve a more comprehensive and systematic knowledge of the tumor immunological microenvironment.

This research made use of public data from the Gene Expression Synthesis (GEO) and a genome Atlas database of early NSCLC to immunotype patients based on the prognostic significance of specific subpopulations of immune cells. We investigated the subtype distribution with regard to clinical features, the significance of prognostic factors, and the subtype-specific heterogeneity-related pathways. Finally, an exploration into the association between subtypes, human immune cell populations, and distinctive genes was carried out. These findings might offer a strong basis for immunotherapy of NSCLC at an early stage in the near future.

## 2. Materials and Methods

### 2.1. The Components and Steps Involved

The RNAseq datasets and subsequent clinical information for NSCLC were collected from the TCGA database (https://www.cancer.gov/about-nci/organization/ccg/research/structural-genomics/tcga). NSCLC covers lung adenocarcinoma (LUAD) and lung squamous cell carcinoma (LUSC). When it comes to the RNAseq data, the genes showing no expression in any of the samples were removed, the expression profile originally presented in FPKM format was changed to TPM format, and log2 processing was performed. The ComBat functionality available in the SVA package [8] was used to combine the LUAD and LUSC samples into a single dataset named RNAseq. This was performed for the purpose of avoiding any batch effects that may have existed between the two different types of samples. The CEL files (including survival time) of the four datasets GSE29013 [9], GSE31210 [10], GSE37745 [11], and GSE50081 [12] were retrieved from the database, and the samples of the GPL570 chip [HG-U133_Plus_2]. The RMA function (robust multiarray average expression way of measuring) of *R* package Affy (V1.66.0) [13] was utilized to process and normalize the expression spectrum data for obtaining the dataset's expression spectrum. It was required to eliminate the batch effect that occurred throughout all four datasets; therefore, the ComBat function included in the SVA package [8] was introduced to combine them all into a single dataset (GSE). We employed the GPL570 annotation file supplied in the combined data set in order to turn the probes into gene symbols. To be more specific, when multiple probes belong to one same gene symbol, the expression spectrum of the gene symbol was taken as its medium value, whereas if a probe corresponded to multiple gene symbols, the expression of the probe was then removed. For the GSE analysis, only samples of early-stage NSCLC with data about survival time and status were kept (Table 1).

For the purpose of gathering clinical data, only samples from stages I and II were retained, while those lacking information regarding survival status or time were deleted. The statistical information regarding the processed samples is presented in Table 2, and in general, the samples were in the early stage of tumor.

The IMMOPORT website, which can be visited at https://www.immport.org/home, was mined for a maximum number of 1793 genes related to immune (Table S1).

### 2.2. Cluster Evaluation

Both the RNAseq dataset and the GSE dataset containing immune genes were subjected to univariate Cox analysis with the coxph function of the *R* package survival (V3.1-12). The *p* value threshold for this analysis was set at 0.05. After that, the genes related to prognosis, which were found at the intersection of the two datasets, were selected for additional study. Next, the *R* package ConsensusClusterPlus (V 1.52.0) was utilized to conduct molecular typing on RNAseq dataset samples and CSE dataset samples in a way distinct from one another [14]. After that, KM curves, which are able to manifest the differences in survival curves, were plotted for the various molecular subtypes. After that, the subtype assignment was confirmed based on the mRNA expression data of the immune genes discussed earlier using the method of t-distribution-based random neighborhood embedding (t-SNE) [15]. In addition, discrepancies in the allocation of clinical features across distinct molecular subtypes were studied using the chi-square test, and a value of *p* < 0.05 was taken to show if there was a statistical difference.

### 2.3. Enrichment of Gene Sets by Analysis Performed on a Single Sample (ssGSEA)

Gene set variation analysis, often known as GSVA, is a method of unattended, nonparametric gene set enrichment that predicts the score of particular pathways or indicators [16]. The method is based on transcriptome data. Previously published study retrieved 16 human signature-related indicator genes [17], the properties of which were known to be linked with tumor drivers and were investigated by using the *R* packages GSVA [16] and GSEABase (V1.50.1). When attempting to quantify immune cell infiltration, the ssGSEA technique is typically applied. The Kruskal–Wallis test was then introduced here in order to compare the scores of immune cell infiltration across the various subtypes.

### 2.4. Analysis of Immune Microenvironment

The immunological microenvironment was analyzed applying the MCP-counter approach [18], which has been considered as being able to accurately quantify the absolute abundant supply of ten distinct cell populations, including eight immune cell populaces (T cells, CD8 T cells, cytotoxic lymphocytes, B lineage, NK cells, and monocytic lineage) as well as two stromal cell populations. The immune cell populations include T cells, CD8 T cells, and NK cells and monocytic lymphocytes (myeloid dendritic cells and neutrophils). The Kruskal–Wallis test was used in order to make a comparison between the scores of immune cell infiltration displayed by the various subtypes. This was conducted for the goal of determining if there was a significant difference between the scores.

The ESTIMATE algorithm was used to assess stromal fractions and immune fractions depending on biomarkers linked with stromal cell and immune cell infiltration in tumor tissues. These fractions were determined based on the stromal fractions and immune fractions. The algorithm estimated stromal fractions and immune fractions with data downloaded from the public website (https://sourceforge.net/projects/estimateproject/). Here, the values of each sample's StromalScore, ImmuneScore, and ESTIMATEScore were calculated via the algorithm of the ESTIMATE [6]. After that, the Kruskal–Wallis test was carried out for the goal of analyzing the subgroups' dissimilarities and similarities.

Twenty-eight immune cell indicators were collected from an earlier work [19], evaluated here with ssGSEA, and the Kruskal–Wallis analysis was carried out to investigate the differences in the score that occurred between the various subtypes of immune cells.

CIBERSORT [20], which was based on the official genes provided for 22 different immune cell types, was used for sample analysis to determine if they included immunological infiltration. After that, the Kruskal–Wallis test was carried out in order to compare the scores of immune cell infiltration that were obtained from the various subtypes.

In addition, immunological checkpoints [21, 22] were selected on the basis of previous research and then evaluated using the Kruskal–Wallis test for distinguishing subtypes more clearly.

### 2.5. Comparison to the Standard of Molecular Typing

TCGA was previously segmented into six distinct immune subtypes, which were designated as follows: IC1 (wound healing), IC2 (INF-*γ* predominant), IC3 (inflammation), IC4 (lymphocyte depletion), and IC5 (immunologically silent) and IC6 (TGF-beta dominant) [23]. After that, a comparison of the molecular subtypes discovered in this research with those previously confirmed in other research studies in the past was conducted.

### 2.6. Tumor Immune Dysfunction and Exclusion

TIDE [24, 25] is an abbreviation that stands for “Tumor Immune Dysfunction and Exclusion.” It is a computational framework that has been designed to evaluate the possibility of immune evasion by malignancies based on the gene expression profiles of cancer samples. TIDE is an acronym for “Tumor Immune Dysfunction and Exclusion.” TIDE gene expression patterns derived from cancer samples have served as the foundation in our analysis. The TIDE score, which is calculated for every tumor sample, could function as a surrogate biomarker to estimate the response to immune checkpoint blockade treatments such as anti-PD1 and anti-CTLA4 when applied to melanoma and NSCLC at an early stage for each tumor sample. TIDE was utilized to predict sample responses inside the RNAseq and GSE datasets. In addition, it was introduced to analyze the proportion of treatment responses among various subtypes and TIDE scores.

### 2.7. Prediction of Patients' Benefits to be Gained from Immunotherapy and Targeted Therapy

Gene pattern [26] category mapping, also known as SubMap, is a technique for comparing similarities between different molecular classes based on the expression profiles of different patient cohorts. This technique was used to determine similarities between the immunotherapy data and the subclasses identified in the previously mentioned dataset.

By anticipating the similarities of differentially expressed genes between immunotherapy-treated participants and untreated participants, researchers could be able to indirectly estimate the efficacy of subimmunotherapy [27, 28] according to the melanoma patients' data with past treatment of immunotherapy.

In addition, pRRophetic [29] was used to predict the IC50 sensitivity of medications such as cisplatin, paclitaxel, sorafenib, erlotinib, and crizotinib in different molecular subtypes.

## 3. Results

### 3.1. Principal Component Analysis

**Figure 1:**
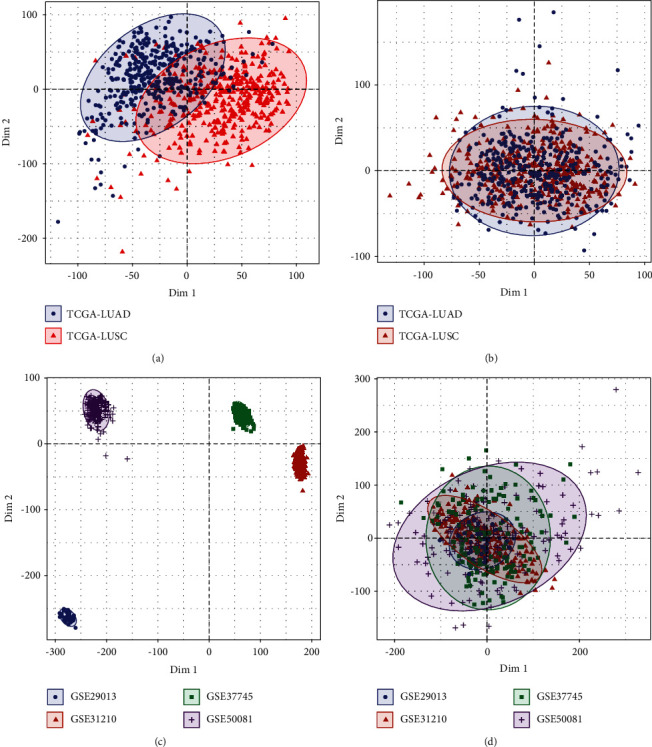
The principal component analysis is shown in this figure (a) A PCA analysis was performed on the RNAseq dataset before the batch effect was removed. (b) PCA analysis of the RNAseq dataset following the elimination of the batch effect. (c) A PCA analysis was performed on the GSE dataset before the batch effect was removed. (d) PCA analysis of the data obtained from the GSE, after the batch effect has been removed.

The workflow chart is showed in Figure S1. The ComBat functionality of the sva package was utilized to execute the TCGA-LUAD and TCGA-LUSC datasets and merge them into a single dataset (RNAseq). In addition, the GSE29013, GSE31210, GSE37745, and GSE50081 datasets were merged into a single dataset. This was carried out for the purpose of excluding the potential presence of the batch effect in a number of different datasets (GSE). PCA allows a clear differentiation between the data obtained from RNAseq (Figures 1(a) and 1(b)) and the GSE dataset (Figures 1(c) and 1(d)) before the batch effect had been removed. However, the data in the dataset were indistinguishable with the existence of the batch effect; in other words, both datasets would be the same.

### 3.2. Using Information from Immunological Profiles, the Identification of Two Distinct Genetic Subtypes of Nonsmall Cell Lung Cancer at a Preliminary Phase

**Figure 2:**
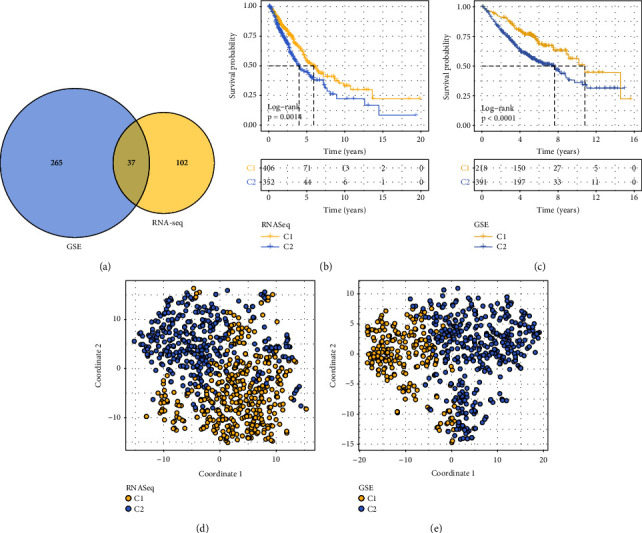
reveals the presence of two distinct molecular subtypes. (a) Venn diagrams comparing the prognostic value of RNAseq and GSE genes. (b) The KM curves for two different subtypes that were taken from the RNAseq dataset. (c) The KM curves for the two different subtypes that are contained inside the GSE dataset. (d) The results of the t-SNE analysis provided support for the hypothesis that RNAseq samples may be divided into two distinct subclasses. (e) The results of the t-SNE analysis provided support for the hypothesis that GSE samples may be divided into two distinct subclasses.

The coxph function of *R* survival was utilized to carry out a univariate cox analysis of genes on the basis of 1,793 immune genes. The coxph function was introduced here in this study for related analysis (V3.1-12). The P filtering threshold was set less than 0.05 for the probability value. The RNAseq dataset had 139 genes associated with prognosis, while the GSE dataset had 302 genes associated with prognosis. Following that, cluster analysis of the subsequent 37 immune-associated prognostic genes was performed using the intersection data of these two sets of data (Figure 2(a)).

After that, the *R* packet ConsensusClusterPlus was used for molecular typing for the samples in the RNAseq dataset and the GSE dataset. The results showed that there were two molecular subclasses, named C1 and C2, in the early-stage NSCLC samples of the RNAseq dataset and the GSE dataset. These molecular subclasses were found in the RNAseq dataset and the GSE dataset.

According to the results of the KM survival analysis, C2 had a less favorable prognosis for survival in comparison to C1 in both the RNAseq and GSE datasets (Figures 2(b) and 2(c)). t-SNE was also utilized to lower the dimension of features in order to confirm the subclass assignment. According to the findings, the subtype names of RNAseq and GSE were, for the most part, similar with two-dimensional t-SNE density variation (Figures 2(d) and 2(e)).

### 3.3. The Relationship Pf Clinical Characteristics of the Two Subtypes

**Figure 3:**
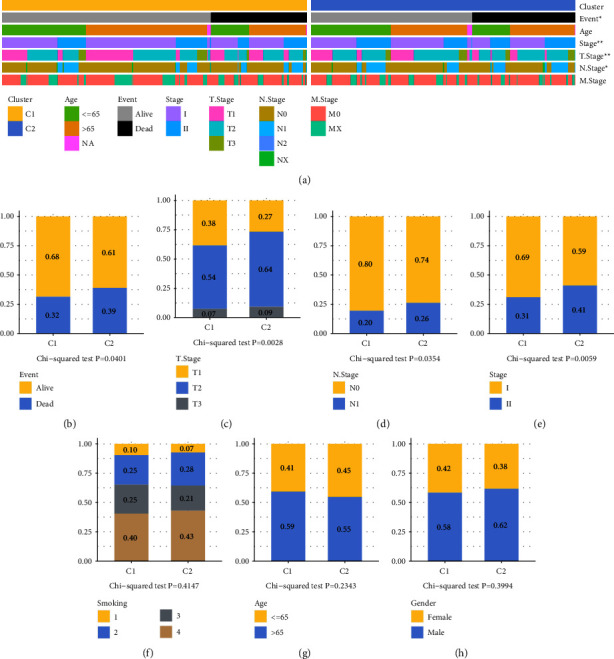
Relationship between immunological subtypes and clinical characteristics (a), such as survival status (b), T stage (c), N stage (d), stages I to IV (e), smoking (f), age (g), and gender (h).

Following this step, the status of survival, age and gender of the individual, *T* phase, *N* phase, *M* phase, smoking history, and stage distributions in the RNAseq dataset were compared between the two molecular subtypes (Figure 3(a)). According to the findings, the ratios of *T* phase, *N* phase, and phase were distinct across the two different molecular subtypes (Figures 3(c)–3(e)), although there were no significant variations in survival status, gender, age, or smoking (Figures 3(a) and 3(e)–3(h)).

### 3.4. Molecular Subtype Immune Infiltration Score Comparison (Immune Microenvironment)

**Figure 4:**
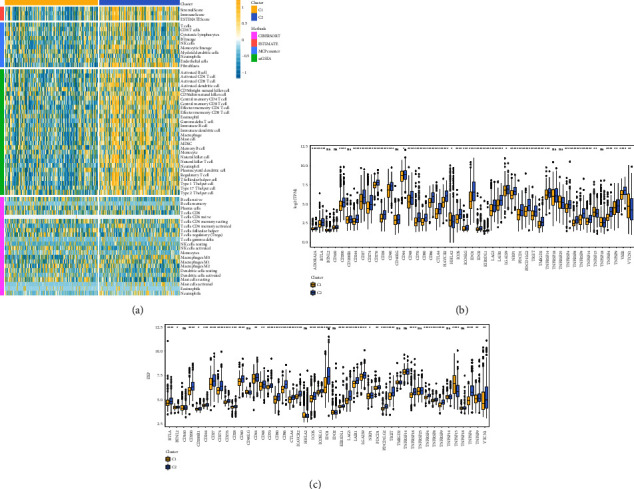
Infiltration of the immune system by two different immunological kinds (Figure 4). (a) A heatmap displaying the immune cell infiltration scores on RNAseq molecular subtypes as assessed by four different applications for immune assessment. (b) A distinction in the levels of expression of immune checkpoint genes between two RNAseq molecular subtypes. Immune checkpoint genes are expressed in a distinct manner in each of the two molecular subtypes of GSE. ^*∗*^*p* less than 0.05, ^*∗∗*^*p* less than 0.01, ^*∗∗∗*^*p* less than 0.001, and ^*∗∗∗∗*^*p* less than 0.0001.

Using MCP-counter, ESTIMATE, ssGSEA, and CIBERSORT, the immune infiltration of the RNAseq dataset samples was analyzed, and the differences in immune cell scores between the two molecular subtypes were compared. The subtype C2, which manifested a relatively poor prognosis and was included in the RNAseq dataset, scored higher in immune infiltration (Figure 4(a)).

In addition, immune checkpoint genes were identified in earlier research [21, 22, 30], and comparisons were made between the two molecular subtypes regarding the variations in the expression levels of these immune checkpoint genes. The findings showed that forty of the forty-seven immunological checkpoints in RNAseq subtypes exhibited significantly variable expression levels. This constituted 85.1 percent of the total (Figure 4(b)). Eighty percent of the forty-five immune checkpoint genes included in the GSE dataset revealed substantial changes in expression among the subtypes (Figure 4(c)). When compared to subtype C1, the expression levels of T cell depletion-related genes CTLA4, PDCD1, and LAG3 were significantly higher in subtype C2 (Figures 4(b) and 4(c)). These findings may potentially explain the phenomena of T cell depletion in subtype C2 as well as the reason why subtype C2 was associated with a high immunological score but had an unfavorable prognosis.

### 3.5. ssGSEA Analysis

**Figure 5:**
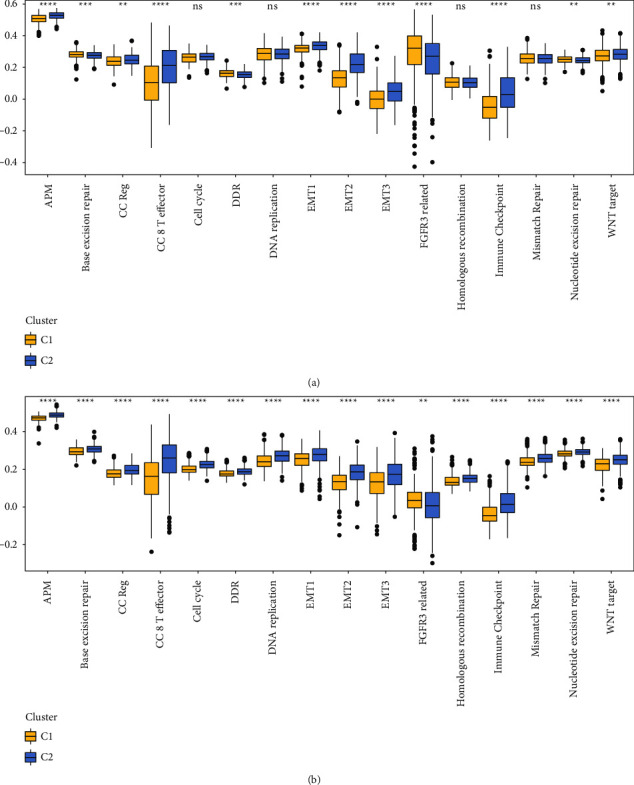
A ssGSEA study of sixteen different human signatures is presented in Figure 5. (a) The difference in ssGSEA scores for 16 human signatures resulting from using two different subtypes of RNAseq. (b) The difference in the ssGSEA score between two different subtypes of GSE for each of the 16 human signatures. Ns means no statistical significance.  ^*∗∗*^*p* less than 0.01,  ^*∗∗∗*^*p* less than 0.001, and  ^*∗∗∗∗*^*p* less than 0.0001.

As part of the GSVA method, 16 human signature- [17] related signals were selected and quantified with the help of ssGSEA, which also facilitates a further examination on the properties of subclasses. These 16 markers showed significant changes between subtypes as well (Figure 5), and these differences were the same in both the RNAseq and the GSE datasets.

### 3.6. Analysis of Similarities and Differences with Previously Identified Immunological Molecular Subtypes

**Figure 6:**
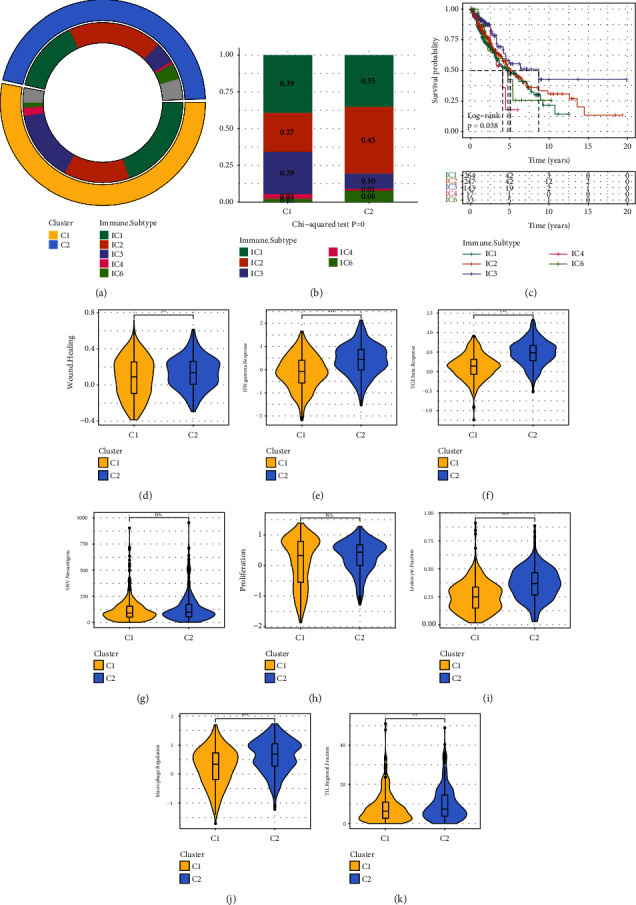
Comparative analysis with previously identified molecular subtypes. (a) A circle graph showing the relationships between the subtypes (The outer layer depicts our molecular subtype, the inner layer illustrates the current immunological typing, and the inner gray circle depicts a sample that has not been identified). (c) KM survival curves for existing molecular subtype distribution of existing molecular subtypes between two immunological subtypes similar to the term “Wound.” Healing (d), IFN.gamma. response (e), response (f), SNV, neoantigens (g), proliferation (h), and leukocyte are all components of TGF-beta. Macrophage, fraction (i), regulation (j), as well as TIL regional, and (k) the fraction of difference between two immune subtypes. Ns means no significance.  ^*∗∗*^*p* less than 0.01,  ^*∗∗*^*p* less than 0.001.

There have been found to be six different kinds of immune invasion in human tumors as a response to tumor-promoting tumor suppressors: IC1 (wound healing), IC2 (INF-R dominant), IC3 (inflammation), IC4 (lymphocyte depletion), IC5 (immunologically silenced), and IC6 (TGF-beta dominant) [23]. Specifically, IC1 refers to the immune system's ability to heal wounds, whereas IC3 refers to inflammation. According to the findings of RNAseq, patients with early-stage NSCLC had the immunological subtypes IC1, IC2, and IC3 (Figure 6(a)). Only 10% of immune subtype IC3 in subtype C2 was linked to a poor prognosis in comparison to 29% of immune subtype IC3 in subtype C1, which was associated with a positive prognosis (Figure 6(b)). The examination of survival curves for these immune subtypes revealed significant differences in OS time between subtypes (Figure 6(c)), with the IC3 immune subtype showing the most favorable prognosis in general.

Additional terms included wound healing, IFN gamma response, TGF beta response, proliferation, and SNV neoantigens. Leukocytes include macrophage, reglementation, and TIL regional. Existing studies also contributed to the current RNAseq data collection [23], based on which the researchers examined the differences that existed between the two subtypes. In addition to SNV neoantigens and proliferation, it has been discovered that there were a number of other traits differing significantly between the two subtypes (Figures 6(d)–6(k)).

### 3.7. TIDE Prediction Response Analysis

Early-stage NSCLC via TIDE projected sample responses in the RNAseq and GSE datasets and could be used in comparing the proportion of treatment responses and TIDE scores for two subgroups of the disease.

**Figure 7:**
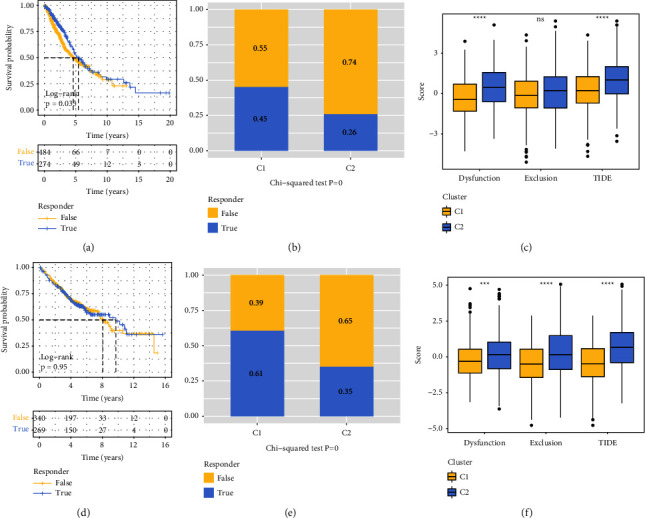
TIDE analysis that predicts the efficacy of immunotherapy across subtypes. (a) The KM curve depicting the TIDE analysis prediction responses in RNAseq. (b) A comparison of the TIDE prediction analysis responses for the two different RNAseq immune subtypes, including the T cell dysfunction score, the T cell exclusion score, and the TIDE score of RNAseq samples. (d) The GSE is the outcome of the KM curve for the TIDE prediction study. (e) A comparison of the findings of the TIDE prediction study performed on the two immunological subtypes of GSE. (f) T cell dysfunction score, T cell exclusion score, and TIDE score of GSE samples. Ns indicates that there is no significant difference between the three scores.  ^*∗∗*^*p* less than 0.01,  ^*∗∗∗*^*p* less than 0.001, and  ^*∗∗∗∗*^*p* less than 0.0001.

The RNASeq dataset contained anticipated response samples that had a better prognosis (Figure 7(a)), and there were statistically significant variations between the various subtypes of responders' response results (Figure 7(b)). In addition, the TIDE score as well as the dysfunction score of the C2 subtype were shown to be higher than those of the C1 subtype (Figure 7(c)). The results obtained from the GSE dataset were the same (Figures 7(d)–7(f)).

### 3.8. Immunotherapy's Effect on the Human Body

**Figure 8:**
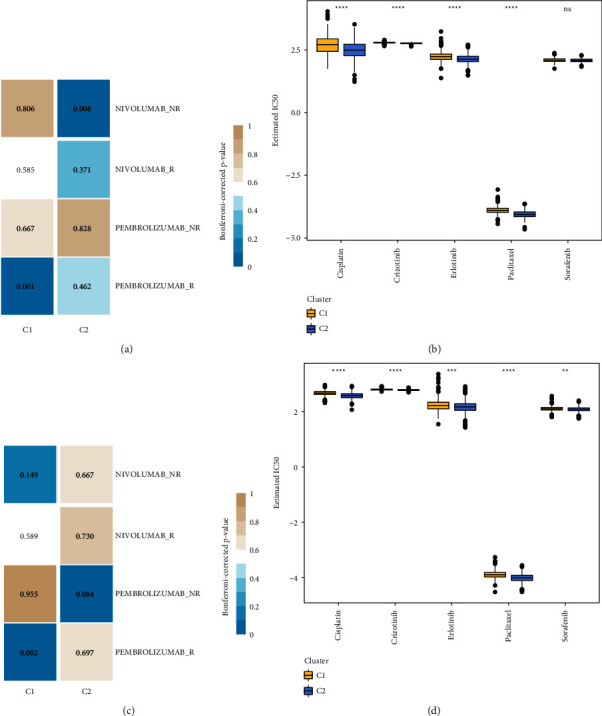
There are three distinct immune subgroups, each of which reacts differently to immunotherapy and chemotherapy. The GSE93157 and RNAseq dataset was analyzed for a submap (a), and the GSE93157 and GSE cohort was analyzed for a submap (b). (c) Estimated concentrations of IC50 for cisplatin, paclitaxel, sorafenib, erlotinib, and crizotinib found in the RNAseq dataset. IC50 values estimated for cisplatin, paclitaxel, sorafenib, erlotinib, and crizotinib in the GSE cohort. Ns indicates that there was no statistical significance.  ^*∗∗*^*p* less than 0.01,  ^*∗∗∗*^*p* less than 0.001, and  ^*∗∗∗∗*^*p* less than 0.0001.

An analytical method known as subclass mapping was introduced here to study the differences between the expression patterns of the two NSCLC subtypes (C1 and C2) in a dataset published in the past and contained patients who had been treated with NIVOLUMAB and PEMBROLIDA (GSE93157 [31]). There was a substantial link between the C1 group and the PEMBROLIZUMAB response group, as shown by the results from RNAseq. Such a result suggested that patients in the C1 group demonstrated a more positive reaction to the therapy with PEMBROLIZUMAB (Figure 8(a)). The GSE dataset presented the exact same occurrence (Figure 8(b)).

In addition, the half-lives, or IC50s, of five different drugs, namely, cisplatin, paclitaxel, sorafenib, erlotinib, and crizotinib, were calculated and compared among the subtypes. These five medical drugs showed substantial differences between the RNAseq dataset (with the exception of sorafenib) and the GSE dataset (Figures 8(c) and 8(d)), and noticeably, the C2 subtype responded more favorably to the therapy than the patients with other subtypes.

## 4. Discussion

Evidence strongly supported that immune cells in the tumor microenvironment have a critical role in the progression of tumors. Tumor-resistant immune cells that are present in the tumor microenvironment have a propensity to hunt and combat cancer cells during the early stages of the development of the tumor. On the other hand, cancer cells are still capable of avoiding immune surveillance and even blocking the function of tumor-resistant immune cells through a variety of different methods for their own survival. This could happen despite the fact that tumor-resistant immune cells in the tumor microenvironment have a tendency to target and kill cancer cells in the early stages of the development of cancer. However, such an unfavorable phenomenon also paves the way for novel cancer therapeutic approaches, such as the use of immune cells to combat cancer cells in the human body. The capacity of cancer to avoid detection of the immune system is a novel sign, and the disease's ability to unlock such detection was not anticipated. Immune checkpoint modulators, such as anti-CTLA4 and anti-PD antibodies, and cultured immune cells such as CAR-T, have shown unexpected antitumor effects across a broad spectrum of cancer types in recent years [32–34]. This has welcomed a new era in the treatment of cancer. However, due to the fact that every cancer patient's tumor microenvironment is unique, anti-PD-1 and anti-PD-L1 therapy is only helpful in the treatment of a small fraction of people with the disease. Discovery of a reliable molecular typing system is the most essential because of its ability to characterize the state of immune cells in the context of making accurate prediction of immunotherapy outcomes. Even though prior research studies [35] were able to identify molecular subtypes for NSCLC in its early stages, immune gene-based molecular typing is still relatively uncommon. In this study, immune-related genes were utilized to classify early-stage NSCLC samples from the RNAseq and GSE datasets into two subgroups (C1 and C2). The C2 subtype was shown to exhibit more immune infiltration and a poorer prognosis than the C1 subtype.

A promising immunotherapy technique for patients with metastatic cancer is the inhibition of immunological checkpoints, such as the PD-1/PD-L1 axis [36]. This strategy involves the suppression of immune checkpoints. T cell fatigue and death can be created when the protein PD-1 generated by T cells interacts with the ligand PD-L1 expressed by immune cells or tumor cells in the tumor microenvironment [37]. It is also possible that the presence of tumor cells is what started such a connection. Our studies have shown that the majority of genes involved in immunological checkpoints appear to have distinct expression patterns between the two subtypes. The markers of T cell exhaustion CTLA4, PDCD1, and LAG3 are all expressed at a high level in C2 cells. Secondly, the results of the TIDE study showed that C2 had the highest scores for T cell dysfunction and exclusion. This suggested that the T cells were malfunctioning as well as had a poor prognosis. In other words, there may be a connection between a high C2 immune escape score and both the inability of T cells to function and the activation of PD-1/PD-L. As a result, we proceeded to investigate the assumption that treatment with anti-PD-1/PD-L1 antibody therapy was successful for C2. It was hypothesized that C2 would benefit greatly from anti-PD-1 inhibitors based on the findings of the subgraph analyses between early-stage NSCLC patient samples and patients treated with NIVOLUMAB and PEMBROLIZUMAB (Figures 8(a) and 8(b)), which was consistent with the findings and hypotheses presented earlier. Therefore, our method of immunological typing may be able to help patients in the early stages of NSCLC and would suggest them to receive individualized treatment for their condition.

The cell cycle, T_CELL_RECEPTOR, B_CELL_RECEPTOR signaling pathways as well as the CHEMOKINE and IMMUNODEFICIENCY signaling pathways are uniquely enriched in one immunological subtype compared to the other. A poor survival among cancer patients is strongly correlated with the activation of immune-related and tumor-related pathways [38–40]. The substantial enrichment of cytokine-cytokine receptor interaction signaling pathways in early-stage NSCLC samples, notably C2, may be able to offer a viable therapeutic target for patients who are in the early stages of NSCLC. In addition, the classification system offered direction for the application of chemotherapeutics in clinical settings. C2 is most sensitive to cisplatin, paclitaxel, sorafenib, and erlotinib among patients in all the subtypes.

Despite the fact that we have used bio-informatics methods on a large sample size to identify two genetic subgroups of early-stage NSCLC with significant prognostic differences, the limitations of our work should be equally noted. In the future, we plan to place a greater emphasis on research that is both fundamentally experimental and functionally in-depth, such as the application of clinical pathological analysis and immunohistochemical expression. Some other considerations were not taken into account because the samples lacked essential clinical follow-up information, most notably diagnostic specifics, for instance, whether the patients had other health conditions in our differentiation of the molecular subtypes.

## 5. Conclusions

In conclusion, we generated two immune subgroups based on genes associated with immune cells to guide tailored therapy for NSCLC patients who were in the early stages of the cancer. Different immune responses were shown by two different immune subgroups in response to chemotherapy and immunotherapy.

## Figures and Tables

**Table 1:** The information of GSE.

GSE	Num	Platform
GSE50081	181	GPL570
GSE29013	38	GPL570
GSE37745	164	GPL570
GSE31210	226	GPL570

**Table 2:** Clinical information of TCGA and GSE.

Feature	TCGA	GSE
Event		
Alive	493	373
Dead	265	236

Gender		
Female	304	
Male	454	

Age		
	425	
≤65	319	
Unknown	14	

T stage		
T1	250	
T2	445	
T3	63	

N stage		
N0	578	
N1	169	
N2	1	
NX	10	

M stage		
M0	577	
MX	181	

Stage		
I	488	449
II	270	160

Smoking		
1	63	
2	195	
3	170	
4	305	
5	7	
7	18	

## Data Availability

All data generated or analyzed during this study are included within this article.

## Supplementary Materials

Supplementary Materials Figure S1: flow chart of work. Table S1: immune genes.
