# Supplementary material for: Early Stage Finding of an Immune-Enhanced Genetic Subtype of Nonsmall Cell Lung Cancer Related with T-Cell Depletion
Source: Evid Based Complement Alternat Med. 2022 Oct 14;2022:6765997. doi: 10.1155/2022/6765997 (PMC9586728; doi:10.1155/2022/6765997)
Supplement: Supplementary Materials — Figure S1: flow chart of work. Table S1: immune genes. [file 6765997.f1.zip › Figure S1.pdf]

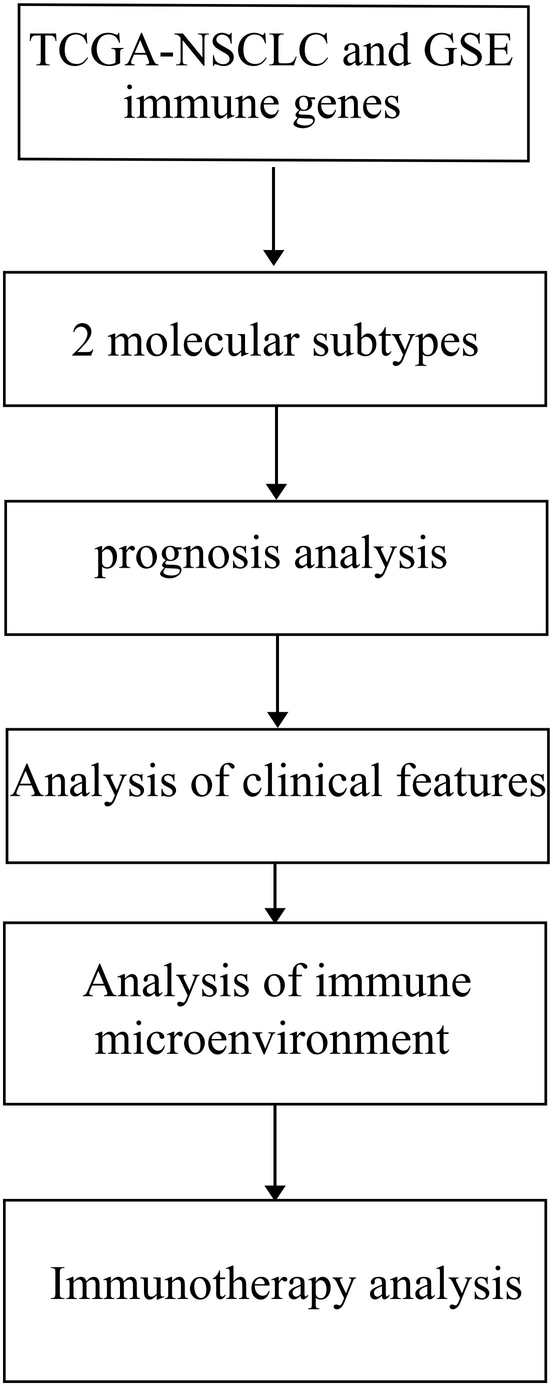
TCGA-NSCLC and GSE immune genes
2 molecular subtypes
prognosis analysis
Analysis of clinical features
Analysis of immune microenvironment
Immunotherapy analysis
