# Supplementary material for: Early Stage Finding of an Immune-Enhanced Genetic Subtype of Nonsmall Cell Lung Cancer Related with T-Cell Depletion
Source: Evid Based Complement Alternat Med. 2022 Oct 14;2022:6765997. doi: 10.1155/2022/6765997 (PMC9586728; doi:10.1155/2022/6765997)
Supplement: Supplementary Materials — Figure S1: flow chart of work. Table S1: immune genes. [file 6765997.f1.zip › Table S1.docx]

**Table S1. immune genes**

AZGP1

B2M

CALR

CANX

CD1A

CD1B

CD1C

CD1D

CD1E

CD4

CD8A

CD8B

CD74

CREB1

CTSB

CTSE

CTSL

CTSS

FCER1G

FCGRT

PDIA3

HFE

HLA-A

HLA-B

HLA-C

HLA-DMA

HLA-DMB

HLA-DOA

HLA-DOB

HLA-DPA1

HLA-DPB1

HLA-DQA1

HLA-DQA2

HLA-DQB1

HLA-DRA

HLA-DRB1

HLA-DRB3

HLA-DRB4

HLA-DRB5

HLA-E

HLA-F

HLA-G

HLA-H

MR1

HSPA1A

HSPA1B

HSPA1L

HSPA2

HSPA4

HSPA5

HSPA6

HSPA8

HSP90AA1

HSP90AB1

ICAM1

IFNA1

IFNA2

IFNA4

IFNA5

IFNA6

IFNA7

IFNA8

IFNA10

IFNA13

IFNA14

IFNA16

IFNA17

IFNA21

IFNG

KIR2DL1

KIR2DL2

KIR2DL3

KIR2DL4

KIR2DS1

KIR2DS3

KIR2DS4

KIR2DS5

KIR3DL1

KIR3DL2

KLRC1

KLRC2

KLRC3

KLRD1

LTA

CIITA

MICA

MICB

NFYA

NFYB

NFYC

LGMN

PSMB8

PSMC1

PSMC2

PSMC3

PSMC4

PSMC5

PSMC6

PSMD1

PSMD2

PSMD3

PSMD4

PSMD5

PSMD7

PSMD8

PSMD10

PSMD11

PSMD13

PSME1

PSME2

RELB

RFX5

RFXAP

SLC10A2

TAP1

TAP2

TAPBP

THBS1

SEM1

KLRC4

AP3B1

RFXANK

PSMD6

PSME3

PSMD14

CLEC4M

IFI30

PROCR

ADRM1

ECPAS

TRPC4AP

CD209

UBXN1

ERAP1

TAPBPL

KIR2DL5A

ERAP2

ULBP3

ULBP2

ULBP1

KIR3DL3

RAET1E

RAET1L

UBR1

RAET1G

PDIA2

HAMP

PI3

CAMP

DEFB4A

PPBP

REG3G

CXCL14

CXCL16

SLPI

CXCL8

CXCL10

CXCL9

CXCL5

CXCL11

CXCL6

CXCL1

CXCL12

CXCL13

CXCL2

PF4

XCL1

CXCL3

DEFB103B

CCL13

CCL1

DEFB1

CCL8

ELANE

DEFB103A

DEFA3

DEFA1

TMSB10

DEFA6

DEFA5

DEFA4

LCN2

LCN1

COLEC10

BPI

S100A9

S100A8

DCD

LCN6

S100A12

HTN3

LCN8

DEFA1B

CCR10

CELA1

DEFB106A

PENK

BPIFC

MMP12

BPIFB6

LEAP2

SFTPD

LCN9

BPIFB2

PTGDS

TMSB4X

PGLYRP1

ZC3HAV1

TMSB15A

S100B

S100A13

S100A6

DEFB119

DEFB107A

DEFB105A

SERPIND1

DEFB129

DEFB127

S100P

S100A7

DEFB104A

DEFB126

DEFB106B

DEFB104B

DEFB107B

PGLYRP3

PGLYRP2

S100A10

S100A2

DEFB125

DEFB123

DEFB105B

DEFB132

BPIFB3

LCN12

PGLYRP4

S100A11

S100A5

S100A3

S100A1

DEFB128

DEFB108B

HTN1

LMBR1L

S100A7A

DEFB118

COLEC12

TMSB4Y

DEFB131A

DEFB134

DEFB130A

DEFB124

DEFB121

DEFB116

DEFB115

DEFB114

DEFB113

DEFB112

DEFB110

TMSB15B

DEFB133

S100Z

MAVS

TMSB4XP8

S100A14

LCN10

S100A16

DEFB136

DEFB135

DEFB117

ZC3HAV1L

S100A7L2

MBL3P

DEFB4B

BPIFB4

IFNAR1

AZU1

DEFB131B

DEFA1A3

LCN1P1

S100G

DEFA7P

DEFB130B

DEFB108F

DEFB131C

TCHHL1

TINAGL1

IFNGR1

SLC22A17

WFIKKN1

WFDC2

IL6

UMODL1

TGFB1

PF4V1

MMP9

ANOS1

TLR4

SPAG11B

A2M

NFKB1

APOBEC3G

FABP6

NOD2

MBL2

SFTPA1

RBP1

TLR2

SLC40A1

PLAU

IL1B

PAEP

HJV

MUC5AC

OBP2A

PLTP

MX1

DDX58

IFNL1

IRF3

SFTPA2

LPA

LBP

RBP4

NOX4

LTF

IFNB1

RBP5

FABP7

FABP5

FABP3

FABP2

FABP4

R3HDML

BPIFA3

BPIFB1

OASL

CRABP2

CRABP1

RBP7

DUOX1

OBP2B

RBP2

LCN15

CETP

FABP12

FABP9

BPIFA1

LCNL1

C8G

SPAG11A

PI15

NOX1

PMP2

APOD

ORM2

ORM1

TNF

CTSG

PRTN3

MAPK1

PML

AEN

CYBB

BPIFA2

ISG20

BCL3

ISG20L2

NOX5

NOX3

DUOX2

TLR3

TFRC

IFIH1

LRP1

TRIM5

IDO1

GDF15

NEDD4

ADIPOQ

STAT3

STAT1

IFNL2

SOCS3

SEMG1

TNFSF10

CCL20

SOCS1

RNASEL

IRF1

IL15

APOBEC3F

PLAAT4

CHIT1

CD40

TLR7

PPIA

ZYX

NLRX1

PGC

VEGFA

IKBKE

ISG15

DHX58

TNFAIP3

TFR2

FCN2

MUC4

F2R

ELN

IL27

MAPT

LYZ

CCL5

LEP

CYLD

KLKB1

CST4

CSRP1

MAPK14

JUN

ITGAV

IRF5

CCR6

IL12B

TLR8

GNLY

CD81

EIF2AK2

APOM

CACYBP

NOD1

MAPK8

MAPK3

BST2

BPHL

PLA2G2A

GRN

NEWENTRY

PDGFRA

GNAI1

WNT5A

FURIN

ADAR

TYK2

NOS2

TRAF3

TPT1

TPM2

NEO1

AHNAK

TLR1

TK2

PRDX2

MX2

FGF2

FGA

TCF7L2

F2RL1

TKFC

MSR1

NFKBIZ

LMBR1

EPPIN

SRC

MPO

ELAVL1

ROBO3

SP1

SOD1

PDF

DLL4

ECD

SLC11A1

DMBT1

STING1

SKIV2L

SEMG2

DES

DCK

DAXX

TNFRSF10A

TNFRSF10B

EED

CCL4

LIMS1

LALBA

APOBEC3H

TMPRSS6

SPINK5

MARCO

BECN1

TNFSF11

KNG1

CSK

KLRK1

KCNH2

JUND

JAK1

CLDN4

CCL28

RNASE3

RN7SL1

IRF7

IREB2

ILK

IL18

IL17A

LTB4R

APOBEC3A

MASP2

TRIM27

RELA

IL7R

IL1A

PTX3

IFNAR2

IFN1@

SYTL1

APOBEC3C

DDX17

PTGS2

HTR1A

SEPTIN7

CD40LG

CD14

MASP1

PROC

MAP2K2

MAP2K1

HRG

NDRG1

IRF9

TRIM22

LANCL1

PPP4C

HMOX1

HMGB1

RNASE7

ABCC4

HGF

HDAC1

IFNLR1

PLSCR1

BACH2

TANK

PIK3CG

ARRB1

RSAD2

STAB2

TBK1

PDYN

PDGFRB

PDCD1

PCSK2

PCSK1

ARG2

AQP9

FASLG

APOH

BIRC5

ANXA6

IL22

VTN

VIM

VCAM1

PRDX1

GFAP

GBP2

ALB

SLC29A3

OAS1

AGER

UNC93B1

TNFSF4

NOS1

ACTG1

ACTA1

ACO1

SERPINA3

CXCR1

CCL15

CCL14

CCL16

CCL19

CCL18

CCL17

CCL26

CCL22

CCR3

CCL4L1

ACKR2

CCR7

CCL27

CCR8

ACKR4

CCL2

CCL21

CCL7

CCL3

CCL11

CCR5

CCL23

CCL25

CCL3L3

CCL4L2

CCL3L1

CCR1

CCL24

XCL2

CXCR4

CXCR6

CCR4

TAFA5

TAFA3

TAFA4

TAFA1

TAFA2

CCL15-CCL14

PTK2B

IL4

CDH1

LTBP1

IL13

IL10

IL2

PPARG

FGR

MIF

CRP

JAK2

PTK2

PTGDR

CD86

HCK

VDR

OLR1

GRK2

TXK

RNASE2

CD79A

CD79B

LYN

SYK

BTK

BLNK

VAV3

VAV1

VAV2

RAC1

RAC2

RAC3

PPP3CA

PPP3CB

PPP3CC

CHP1

PPP3R1

PPP3R2

CHP2

NFAT5

NFATC1

NFATC2

NFATC3

NFATC4

HRAS

KRAS

NRAS

FOS

CARD11

BCL10

MALT1

CHUK

IKBKB

IKBKG

NFKBIA

NFKBIB

NFKBIE

CD19

CR2

PIK3R5

PIK3R1

PIK3R2

PIK3R3

PIK3CA

PIK3CB

PIK3CD

AKT3

AKT1

AKT2

GSK3B

INPP5D

CD22

CD72

PTPN6

LILRB3

FCGR2B

RASGRP3

PLCG2

PRKCB

IFITM1

IGH

IGHA1

IGHA2

IGHD

IGHD1-1

IGHD1-14

IGHD1-20

IGHD1-26

IGHD1-7

IGHD2-15

IGHD2-2

IGHD2-21

IGHD2-8

IGHD3-10

IGHD3-16

IGHD3-22

IGHD3-3

IGHD3-9

IGHD4-11

IGHD4-17

IGHD4-23

IGHD4-4

IGHD5-12

IGHD5-18

IGHD5-24

IGHD5-5

IGHD6-13

IGHD6-19

IGHD6-25

IGHD6-6

IGHD7-27

IGHE

IGHG1

IGHG2

IGHG3

IGHG4

IGHJ1

IGHJ2

IGHJ3

IGHJ4

IGHJ5

IGHJ6

IGHM

IGHV1-18

IGHV1-2

IGHV1-24

IGHV1-3

IGHV1-45

IGHV1-46

IGHV1-58

IGHV1-69

IGHV1-8

IGHV1-38-4

IGHV1-69-2

IGHV2-26

IGHV2-5

IGHV2-70

IGHV3-11

IGHV3-13

IGHV3-15

IGHV3-16

IGHV3-20

IGHV3-21

IGHV3-23

IGHV3-30

IGHV3-30-3

IGHV3-30-5

IGHV3-33

IGHV3-35

IGHV3-38

IGHV3-43

IGHV3-48

IGHV3-49

IGHV3-53

IGHV3-64

IGHV3-66

IGHV3-7

IGHV3-72

IGHV3-73

IGHV3-74

IGHV3-9

IGHV3-38-3

IGHV3-69-1

IGHV4-28

IGHV4-30-1

IGHV4-30-2

IGHV4-30-4

IGHV4-31

IGHV4-34

IGHV4-39

IGHV4-4

IGHV4-59

IGHV4-61

IGHV4-38-2

IGHV5-51

IGHV5-10-1

IGHV6-1

IGHV7-4-1

IGHV7-81

IGK

IGKC

IGKDEL

IGKJ

IGKJ1

IGKJ2

IGKJ3

IGKJ4

IGKJ5

IGKV@

IGKV1-12

IGKV1-13

IGKV1-16

IGKV1-17

IGKV1-27

IGKV1-33

IGKV1-37

IGKV1-39

IGKV1-5

IGKV1-6

IGKV1-8

IGKV1-9

IGKV1D-12

IGKV1D-13

IGKV1D-16

IGKV1D-17

IGKV1D-33

IGKV1D-37

IGKV1D-39

IGKV1D-42

IGKV1D-43

IGKV1D-8

IGKV2-24

IGKV2-28

IGKV2-30

IGKV2-40

IGKV2D-24

IGKV2D-28

IGKV2D-29

IGKV2D-30

IGKV2D-40

IGKV3-11

IGKV3-15

IGKV3-20

IGKV3-7

IGKV3D-11

IGKV3D-15

IGKV3D-20

IGKV3D-7

IGKV4-1

IGKV5-2

IGKV6-21

IGKV6D-21

IGKV6D-41

IGL

IGLC1

IGLC2

IGLC3

IGLC6

IGLC7

IGLJ

IGLJ1

IGLJ2

IGLJ3

IGLJ4

IGLJ5

IGLJ6

IGLJ7

IGLV@

IGLV1-36

IGLV1-40

IGLV1-44

IGLV1-47

IGLV1-50

IGLV1-51

IGLV10-54

IGLV11-55

IGLV2-11

IGLV2-14

IGLV2-18

IGLV2-23

IGLV2-33

IGLV2-8

IGLV3-1

IGLV3-10

IGLV3-12

IGLV3-16

IGLV3-19

IGLV3-21

IGLV3-22

IGLV3-25

IGLV3-27

IGLV3-32

IGLV3-9

IGLV4-3

IGLV4-60

IGLV4-69

IGLV5-37

IGLV5-39

IGLV5-45

IGLV5-48

IGLV5-52

IGLV6-57

IGLV7-43

IGLV7-46

IGLV8-61

IGLV9-49

C3

C5

CCL3P1

CKLF

CMA1

CX3CL1

CXCL17

CCN1

EDN1

EDN2

EDN3

FGF10

LECT2

PPBPP1

PROK2

SAA1

SAA2

SBDS

SEMA3A

SEMA3B

SEMA3C

SEMA3D

SEMA3E

SEMA3F

SEMA3G

SEMA4A

SEMA4B

SEMA4C

SEMA4D

SEMA4F

SEMA4G

SEMA5A

SEMA5B

SEMA6A

SEMA6B

SEMA6C

SEMA6D

SEMA7A

SLIT1

SLIT2

TNC

TYMP

C5AR1

CCR9

CCRL2

CMKLR1

CX3CR1

CXCR3

CXCR5

ACKR3

CYSLTR1

CYSLTR2

ACKR1

EDNRA

EDNRB

FPR1

FPR2

GPR17

GPR32

GPR33

PTGDR2

C5AR2

CXCR2

LTB4R2

PLAUR

PLXNA1

PLXNA2

PLXNA3

PLXNA4

PLXNB1

PLXNB2

PLXNB3

PLXNC1

PLXND1

PTAFR

ROBO1

ROBO2

RXFP3

XCR1

ADM

ADM2

AGRP

AGT

AMBN

AMELX

AMH

ANGPTL5

ANGPTL7

APLN

AREG

MANF

CDNF

ARTN

AVP

BDNF

BMP1

BMP10

BMP15

BMP2

BMP3

BMP4

BMP5

BMP6

BMP7

BMP8A

BMP8B

BTC

MYDGF

CALCA

CALCB

CAT

CCK

CD320

CD70

ADA2

CER1

CGA

CGB3

CGB1

CGB2

CGB5

CGB7

CGB8

CHGA

CHGB

CLCF1

CLEC11A

CMTM1

CMTM2

CMTM3

CMTM4

CMTM5

CMTM6

CMTM7

CMTM8

CNTF

CORT

CRH

CSF1

CSF2

CSF3

CSH1

CSH2

CSHL1

CSPG5

CTF1

CCN2

DKK1

EBI3

EGF

EPGN

EPO

EREG

ESM1

FAM3B

FAM3C

FAM3D

FGF1

FGF11

FGF12

FGF13

FGF14

FGF16

FGF17

FGF18

FGF19

FGF20

FGF21

FGF22

FGF23

FGF3

FGF4

FGF5

FGF6

FGF7

FGF8

FGF9

VEGFD

FIGNL2

FLT3LG

FSHB

GAL

GALP

GAST

GCG

GDF1

GDF10

GDF11

GDF2

GDF3

GDF5

GDF6

GDF7

GDF9

GDNF

GH1

GH2

GHRH

GHRL

GIP

GKN1

GMFB

GMFG

GNRH1

GNRH2

GPHA2

GPHB5

GPI

GREM1

GREM2

GRP

GUCA2A

HBEGF

HDGF

HDGFL3

IAPP

IFNE

IFNK

IFNW1

IGF1

IGF2

IL11

IL12A

IL16

IL17B

IL17C

IL17D

IL17F

IL19

IL1F10

IL36RN

IL36A

IL37

IL36B

IL36G

IL1RN

IL20

IL21

IL23A

IL24

IL25

IL26

IFNL3

IL3

IL31

IL32

IL33

IL34

IL5

IL6ST

IL7

IL9

INHA

INHBA

INHBB

INHBC

INHBE

INS

INS-IGF2

INSL3

INSL4

INSL5

INSL6

JAG1

JAG2

FGF7P6

FGF7P3

KITLG

KL

LACRT

LEFTY1

LEFTY2

LHB

LIF

LRSAM1

LTB

LTBP2

LTBP3

LTBP4

MDK

MIA

MLN

MSTN

NAMPT

NDP

NENF

NGF

NMB

NODAL

CCN3

NPFF

NPPA

NPPB

NPPC

NPY

NRG1

NRG2

NRG3

NRG4

NRTN

NTF3

NTF4

NTS

NUDT6

OGN

OSGIN1

OSM

OSTN

OXT

ENDOU

PDGFA

PDGFB

PDGFC

PDGFD

PDGFRL

PGF

PMCH

PNOC

POMC

PPBPP2

PPY

PRL

PRLH

PROK1

PSPN

PTH

PTH2

PTHLH

PTN

PYY

QRFP

RABEP1

RABEP2

REG1A

RETN

RETNLB

RLN1

RLN2

RLN3

SCG2

SCGB3A1

SCT

AIMP1

SECTM1

SLURP1

SPP1

SST

STC1

STC2

TAC1

TDGF1

TDGF1P3

TG

TGFA

TGFB2

TGFB3

THPO

TNFRSF11B

TNFSF12

TNFSF13

TNFSF13B

TNFSF14

TNFSF15

TNFSF18

TNFSF8

TNFSF9

TOR2A

TRH

TSHB

TSLP

TXLNA

UCN

UCN2

UCN3

UTS2

UTS2B

VEGFB

VEGFC

VGF

VIP

ACVR1B

ACVR1C

ACVR2A

ACVR2B

ACVRL1

ADCYAP1R1

ADIPOR1

ADIPOR2

ADRB1

ADRB2

AGTR1

AGTR2

AMHR2

ANGPT1

ANGPT4

ANGPTL1

ANGPTL2

ANGPTL3

ANGPTL4

ANGPTL6

APLNR

AR

AVPR1A

AVPR1B

AVPR2

BMPR1A

BMPR1B

BMPR2

BRD8

C3AR1

CALCR

CALCRL

CNTFR

CRHR1

CRHR2

CRIM1

CRLF1

CRLF2

CRLF3

CSF1R

CSF2RA

CSF2RB

CSF3R

EGFR

ENG

EPOR

ESR1

ESR2

ESRRA

ESRRB

ESRRG

FGFR1

FGFR2

FGFR3

FGFR4

FGFRL1

FLT1

FLT3

FLT4

FSHR

GALR2

GALR3

GCGR

GHR

GHRHR

GHSR

GIPR

GLP1R

GLP2R

GNRHR

GPER1

HNF4A

HNF4G

HTR3A

HTR3B

HTR3C

HTR3D

HTR3E

IFNGR2

IGF1R

IGF2R

IL10RA

IL10RB

IL11RA

IL12RB1

IL12RB2

IL13RA1

IL13RA2

IL15RA

IL2RB

IL17RA

IL17RB

IL17RC

IL17RD

IL17RE

IL18R1

IL18RAP

IL1R1

IL1R2

IL1RAP

IL1RL1

IL1RL2

IL20RA

IL20RB

IL21R

IL22RA1

IL22RA2

IL23R

IL27RA

IL2RA

IL2RG

IL31RA

IL3RA

IL4R

IL5RA

IL6R

IL9R

INSR

KDR

LEPR

LGR4

LGR5

LGR6

LHCGR

LIFR

LTBR

MC1R

MC2R

MC3R

MC4R

MCHR1

MCHR2

MET

MLNR

MPL

MTNR1A

MTNR1B

NGFR

NMBR

NPR1

NPR3

NR0B1

NR0B2

NR1D1

NR1D2

NR1H2

NR1H3

NR1H4

NR1I2

NR1I3

NR2C1

NR2C2

NR2E1

NR2E3

NR2F1

NR2F2

NR2F6

NR3C1

NR3C2

NR4A1

NR4A2

NR4A3

NR5A1

NR5A2

NR6A1

NRP1

NRP2

OGFR

OPRD1

OPRK1

OPRL1

OPRM1

OSMR

OXTR

PGR

PGRMC2

PPARA

PPARD

PRLHR

PRLR

PTGER1

PTGER2

PTGER3

PTGER4

PTGFR

PTH1R

PTH2R

RARA

RARB

RARG

RORA

RORB

RORC

RXFP1

RXFP2

RXRA

RXRB

RXRG

S1PR1

S1PR2

SCTR

SDC1

SDC2

SDC3

SDC4

SORT1

SSTR1

SSTR2

SSTR5

ST2

TACR1

TEK

TGFBR1

TGFBR2

TGFBR3

THRA

THRB

TIE1

TNFRSF10C

TNFRSF10D

TNFRSF11A

TNFRSF12A

TNFRSF13B

TNFRSF13C

TNFRSF14

TNFRSF17

TNFRSF18

TNFRSF19

TNFRSF1A

TNFRSF1B

TNFRSF21

TNFRSF25

TNFRSF4

TNFRSF6B

TNFRSF8

TNFRSF9

TRHR

TSHR

TUBB3

VIPR1

VIPR2

PTPN11

ICAM2

ITGAL

ITGB2

PAK1

NCR2

TYROBP

LCK

FCGR3A

FCGR3B

NCR1

NCR3

CD247

ZAP70

LCP2

LAT

PLCG1

SH3BP2

FYN

SHC2

SHC4

SHC3

SHC1

GRB2

SOS1

SOS2

ARAF

BRAF

RAF1

HCST

CD48

CD244

PRKCA

PRKCG

SH2D1B

SH2D1A

FAS

GZMB

PRF1

CASP3

BID

CD3D

CD3E

CD3G

PTPRC

ITK

TEC

NCK1

NCK2

GRAP2

PAK2

PAK3

PAK4

PAK6

PAK5

RHOA

CDC42

CD28

ICOS

MAP3K8

MAP3K14

CTLA4

CBLC

CBL

CBLB

CDK4

RASGRP1

PDK1

PRKCQ

TRAC

TRAJ1

TRAJ2

TRAJ3

TRAJ4

TRAJ5

TRAJ6

TRAJ7

TRAJ8

TRAJ9

TRAJ10

TRAJ11

TRAJ12

TRAJ13

TRAJ14

TRAJ15

TRAJ16

TRAJ17

TRAJ18

TRAJ19

TRAJ20

TRAJ21

TRAJ22

TRAJ23

TRAJ24

TRAJ25

TRAJ26

TRAJ27

TRAJ28

TRAJ29

TRAJ30

TRAJ31

TRAJ32

TRAJ33

TRAJ34

TRAJ35

TRAJ36

TRAJ37

TRAJ38

TRAJ39

TRAJ40

TRAJ41

TRAJ42

TRAJ43

TRAJ44

TRAJ45

TRAJ46

TRAJ47

TRAJ48

TRAJ49

TRAJ50

TRAJ52

TRAJ53

TRAJ54

TRAJ56

TRAJ57

TRAJ58

TRAJ59

TRAJ61

TRAV1-1

TRAV1-2

TRAV2

TRAV3

TRAV4

TRAV5

TRAV7

TRAV8-1

TRAV8-2

TRAV8-3

TRAV8-4

TRAV8-6

TRAV8-7

TRAV9-1

TRAV9-2

TRAV10

TRAV12-1

TRAV12-2

TRAV12-3

TRAV13-1

TRAV13-2

TRAV14DV4

TRAV16

TRAV17

TRAV18

TRAV19

TRAV20

TRAV21

TRAV22

TRAV23DV6

TRAV24

TRAV25

TRAV26-1

TRAV26-2

TRAV27

TRAV29DV5

TRAV30

TRAV34

TRAV35

TRAV36DV7

TRAV38-1

TRAV38-2DV8

TRAV39

TRAV40

TRAV41

TRBC1

TRBC2

TRBD1

TRBD2

TRBJ1-1

TRBJ1-2

TRBJ1-3

TRBJ1-4

TRBJ1-5

TRBJ1-6

TRBJ2-1

TRBJ2-2

TRBJ2-3

TRBJ2-4

TRBJ2-5

TRBJ2-6

TRBJ2-7

TRBV2

TRBV3-1

TRBV4-1

TRBV4-2

TRBV4-3

TRBV5-1

TRBV5-4

TRBV5-5

TRBV5-6

TRBV5-7

TRBV5-8

TRBV6-1

TRBV6-2

TRBV6-3

TRBV6-4

TRBV6-5

TRBV6-6

TRBV6-7

TRBV6-8

TRBV6-9

TRBV7-2

TRBV7-3

TRBV7-4

TRBV7-6

TRBV7-7

TRBV7-8

TRBV7-9

TRBV9

TRBV10-1

TRBV10-2

TRBV10-3

TRBV11-1

TRBV11-2

TRBV11-3

TRBV12-3

TRBV12-4

TRBV12-5

TRBV13

TRBV14

TRBV15

TRBV16

TRBV17

TRBV18

TRBV19

TRBV20-1

TRBV24-1

TRBV25-1

TRBV27

TRBV28

TRBV29-1

TRBV30

TRDC

TRDD1

TRDD2

TRDD3

TRDJ1

TRDJ2

TRDJ3

TRDJ4

TRDV1

TRDV2

TRDV3

TRGV9

TRGV8

TRGV5

TRGV4

TRGV3

TRGV2

TRGJP2

TRGJP1

TRGJP

TRGJ2

TRGJ1

TRGC2

TRGC1

TRAV6
